# Protocol for Streaming Data from an RFID Sensor Network †

**DOI:** 10.3390/s19143148

**Published:** 2019-07-17

**Authors:** Gentza Souto, Florian Muralter, Laura Arjona, Hugo Landaluce, Asier Perallos

**Affiliations:** DeustoTech, University of Deusto, 48940 Bilbao, Spain

**Keywords:** RFID sensor network, data streaming, wireless sensor network, EPC C1G2

## Abstract

Currently, there is an increasing interest in the use of Radio Frequency Identification (RFID) tags which incorporate passive or battery-less sensors. These systems are known as computational RFID (CRFID). Several CRFID tags together with a reader set up an RFID sensor network. The reader powers up the tags’ microcontroller and their attached sensor using radio frequency waves, and tags backscatter, not only their EPC code but also the value of those sensors. The current standard for interrogating these CRFID tags is the EPC global Class 1 Generation 2 (EPC C1G2). When several tags are located inside the reader interrogation area, the EPC C1G2 results in very poor performance to obtain sensor data values. To solve this problem, a novel protocol called Sensor Frmed Slotted Aloha (sFSA) for streaming sensor data dealing with the tag collisions is presented. The proposed protocol increases the Sensor Read Rate (SRR), defined as the number of sensor data reads per second, compared to the standard. Additionally, this paper presents a prototype of an RFID sensor network to compare the proposed sFSA with the standard, increasing the SRR by more than five times on average. Additionally, the proposed protocol keeps a constant sensor sampling frequency for a suitable streaming of these tag sensors.

## 1. Introduction

The direct adaptability of Radio Frequency Identification (RFID) to the demands of the supply chain is continuously increasing this technologies’ popularity. Goods tracking and monitoring, traceability of patients or managing the medication of elderly people in health-care are a number of applications that have benefited from using RFID [1].

A typical RFID system is composed of an interrogator (reader) and at least one transponder (tag). The reader interrogates the tags by transmitting radio frequency (RF) signals. Tags are devices that can be either active or passive and will respond with their Electronic Product Code (EPC). Active tags are powered by batteries, whilst passive tags use the reader signal to power up their circuitry. The latter devices are becoming more and more attractive due to their low cost (≈10 cents), a small sticker form factor, and the factor that battery-life is no longer limiting the tags’ lifetime. Another upcoming area within RFID is computational RFID (CRFID) systems [2]. Several research applications have been proposed for environmental monitoring [3], activity recognition [4] or the battery-free camera [5]. These systems use passive tags containing a programmable micro-controller and a sensor. These sensor tags are powered using the reader RF signal and respond by backscattering their EPC code and the current value of the sensor.

**Figure 1:**
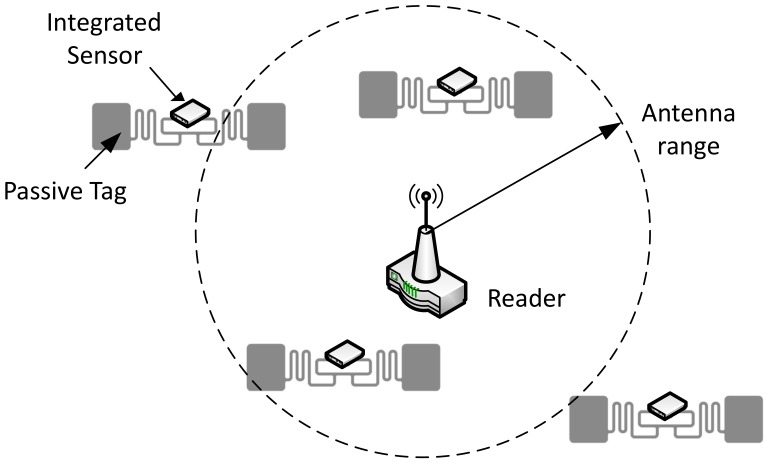
Example of a Radio Frequency Identification (RFID) sensor network [8].

Several passive sensor tags together with a reader create an RFID wireless sensor network (see Figure 1), which offers the possibility of densely distributed sensing, inheriting the mentioned advantages of RFID. Various solutions were proposed that merge RFID technology with Wireless Sensor Networks (WSN) [6,7] to improve the capabilities and the applicability of either technology. One way of combining RFID and WSNs is the incorporation of sensors in RFID tags.

One of the main problems an RFID system faces is the existence of various tags transmitting at the same time in the range of the same reader. As tags share the transmission channel, their responses may not be decoded correctly as a result of interfering waves. This well-known problem is called the tag collision problem [9,10,11] and forces tags to re-transmit the tag messages until the reader identifies them correctly by receiving their unique ID code. A medium access control (MAC) protocol is needed to overcome this issue. Within the field of RFID this is named anti-collision protocol due to the particularities the technology adds to the problem [11].

The main focus of this paper is on a CRFID WSN, with one reader, which interrogates several CRFID tags. Usually, these types of sensor networks use the same protocols for reading the tag’s EPC and collecting the sensed data [12]. This fact makes the sensing process very inefficient because the reader has to face the tag collision problem first, identify the tag in the range of the antenna, and then collect its sensed data from the passive sensors. This process is then repeated for every requested dataset.

The current standard used in RFID is the EPCglobal Class 1 Gen 2 protocol (EPC C1G2) [13]. This is an arbitration oriented protocol used in every commercial reader, also included in ISO 18000-6C. The main purpose of this protocol is not the reading of sensor data from a CRFID tag. Receiving data from a sensor tag requires a high overhead and can always be handicapped by it not being read.

In order to improve the performance of the standard EPC C1G2 when streaming sensor data from passive sensors, the sensor Frame Slotted Aloha protocol (sFSA) is proposed. This work analyzes the times consumed by the identification and the read phase and, taking into account this analysis, proposes the sFSA protocol. Furthermore, it presents an experimental performance evaluation of an RFID sensor network using CRFID tags using the proposed sFSA protocol in comparison with the standard EPC C1G2 [13]. These measurements are carried out using a physical platform which consists of a software defined radio (SDR) reader and several Wireless Identification and Sensing Platform (WISP) tags [2].

The WISP is a battery-free RFID sensor device powered via the RF energy transmitted by the reader. It carries a programmable micro-controller that allows the use of different identification and sensing protocols. Furthermore, these tags contain an accelerometer, whose measurement data can be written into the user memory and read via the RFID communication. The analysis of the implementations performed within this work show that the proposed protocol increases the Sensor Read Rate (SRR), defined as the number of sensor data reads per second, compared to EPC C1G2 by more than five times on average.

This paper represents an extension of [8] published in the proceedings of the 12th International Conference on Ubiquitous Computing and Ambient Intelligence—UCAmI 2018. This extended version incorporates an approach to solve the problem of non-unique handles considering the proposed protocol, closer describes the implemented protocol and platform, and presents an expanded experimentation section.

The paper is organized using the following structure: Section 2 presents the background and the related work; Section 3 shows how the proposed sFSA works; Section 4 presents the implementation of the tested protocols; Section 5 contains the experimentation details and the performance analysis; and Section 6 concludes this work and proposes future work.

## 2. Background

First, the main parameters used in the paper are properly defined to set the background:

- Sensor tag: RFID tag which incorporates a sensor.
- Tag identification: process to read the tag identifier or the EPC stored in the tag’s memory.
- Slot: period of time that separates the tags’ responses. Conventionally, three types of slots can occur: single (only one tag replies), collision (more than one tag replies in the same slot), and idle (none of the tags reply).
- Frame: sequence of slots. An inventory round is composed of a set of frames and tags can respond in only one slot per frame.
- Inventory round: the period of time that begins when the reader transmits the initial command, and ends when the reader interrupts the Identification Phase.
- Reading round: the period of time that is used to receive the data from the sensor tags identified during the inventory round. This round is usually determined by a number of data samples to be collected from each sensor.

### 2.1. Related Work

Several approaches that solve the tag collision problem and additionally allow to collect sensor data have been proposed. Their methodologies can be subdivided into two categories:

1. Focused on the anti-collision problem. The main existing anti-collision protocols can be classified into tree-based and aloha-based protocols. Tree-based protocols [14] split colliding tags into subsets, and further split the subsets repeatedly until the successful response of all tags within the interrogation zone. Aloha-based protocols [15], on the other hand, divide the time into slotted frames, where tags can only respond once per frame in a randomly chosen slot. The most popular aloha-based protocol is the Dynamic Frame Slotted Aloha used in the standard EPC C1G2 [13]. These protocols are relatively slow due to tag-collisions and the need to go through the inventory round to read sensor data. This increases the occupancy of the wireless channel and thus, they are not recommended to be used for highly time restricted applications.
2. Focused on Wireless Sensor Networks (WSN) based on RFID [6,7]. These types of protocols [16,17] typically assume that all tag IDs are known to the system in advance. However, this assumption is not always true, since dynamic scenarios are very popular among RFID systems. Thus, unexpected replies will severely affect the reading performance. There are other alternatives such as [18] which consider this problem, however, physical implementations have not been provided to the author’s knowledge.

The proposed protocol presents a modification of the standard EPC C1G2, which is an anti-collision problem focused protocol. As it has been explained above, these protocols need to perform an inventory round every time they need to receive the sensors’ values. The proposed modification performs this inventory round phase only once, and then continuously executes the sensing phase, achieving higher reading rates, which makes it more suitable for streaming purposes. To better understand the benefits of the contribution, the EPC C1G2 is first presented before the explanation of the main contribution.

### 2.2. EPC Class 1 Generation 2 Standard

The EPC C1G2 is the standard used in UHF RFID systems [13]. This standard contains the different layers that define the communication between reader and tag and thus, includes the Identification Phase (inventory round) and the Sense phase (reading round).

**Figure 2:**
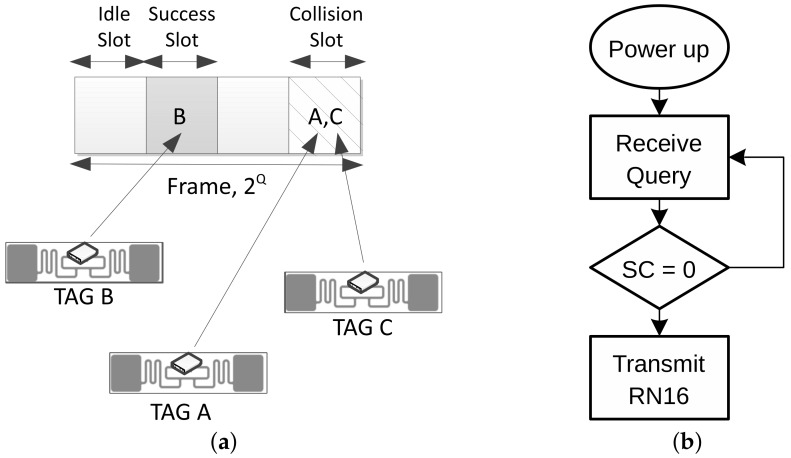
EPC C1G2 definition and slot selection process [8]. (**a**) Slot definitions for EPC C1G2 Identification Phase; (**b**) Tag response procedure to Query command.

1. The Identification Phase. During this phase, the reader tries to obtain the EPC, which uniquely identifies each particular tag. EPC C1G2 uses a Dynamic Frame Slotted Aloha (DFSA) protocol to arbitrate tags’ collisions [13]. It follows a Time Division Multiple Access (MAC) approach, scheduling the tags’ responses along time slots (see Figure 2a). The Identification process begins with the transmission of a *Query* command to set the frame size to a value of $2^{Q}$. Tags randomly select a slot within the frame. This initial value of their internal slot counter SC ranging from 0 to $2^{Q}-1$ is decremented with every slot change. The tag then responds to the reader when SC=0, as stated in Figure 2b. Waiting tags decrease their counter every time the reader transmits a *Query* command to jump to the next slot. If tags’ responses collide, the tags will wait until the next frame, choose a different slot and re-transmit their message. The probability of collisions is sensitive to the choice of the frame size, whose optimum setting depends on the number of responding tags (typically unknown to the reader). Thus, when SC = 0, the tag transmits a 16-bit random number (RN16). Once it is acknowledged (ACK), the tag transmits its unique EPC code. The Identification Phase ends when the reader has received and correctly decoded the EPC from each unique tag. The reader command flow to identify one tag would be as follows (assuming no collision occurred): *Query*-*ACK*. The higher the frame size with respect to the number of tags in the interrogation zone, the higher the number of idle slots that will occur. On the contrary, the lower the frame size, the higher the number of collided slots. According to [11], the optimal frame size is equal to the number of tags located in the reading range.
2. The Sense phase. This time period starts once a particular tag has been identified. During the Sense phase the reader reads data from the sensor tag. To do so, the reader transmits a *Req_RN16* command, requesting a smaller bit-string (16 bits), named handle, from the tag. The handle typically expires after the reading process. The read phase ends when the reader has received one dataset from the tag. The reader command flow during the Sense phase is: *Req_RN16*(RN16)-*Read*(handle).

**Figure 3:**
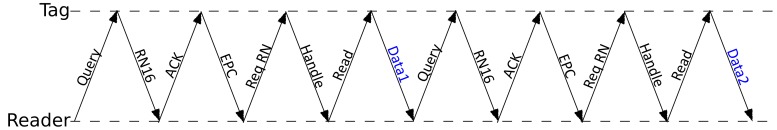
Example of the procedure followed by the standard protocol for *N* = 2 and *S* = 2.

Figure 3 shows an example of the procedure followed by the reader to obtain two sensor reads (S=2) from two tags (N=2), using the standard EPC C1G2. The reader commands flow would be as follows (assuming no collision occurred): *Query*-*ACK*(RN16)-*Req_RN16*(RN16)-*Read*(handle 1)-*Query*-*ACK*(RN16)-*Req_RN16*(RN16)-*Read*(handle 2).

It can be illustrated that the reader must receive the EPC and handle from a sensor tag before transmitting a Read command to read additional sensor data. This paper proposes a different approach to improve the efficiency of sensors’ data streaming, by avoiding the overhead caused by having to identify again each sensor tag before reading additional sensor data.

## 3. The Sensor Frame Slotted Aloha (sFSA)

Using the EPC C1G2, the reader is forced to identify a tag before reading its sensor. This procedure results in a very inefficient strategy for streaming data from sensors. The proposed custom protocol for streaming sensor data provides a much higher reading rate than the protocol used in the current RFID standard. It slightly modifies the Identification Phase of the EPC C1G2 extending it until the reception of at least one handle bit-string of the available tags in the antenna range. This handle is related to the EPC received, and thus, the reader is able to read data from all the sensors by using only these 16 bit handles, instead of their EPCs.

The proposed protocol employs the interrogation and access commands from EPC C1G2. It is therefore compatible with sensor tags following the EPC C1G2 standard, with the particularity that tags must remember their last transmitted handle until they lose their state. The presented custom protocol is also separated into two phases: Identification and Sense Phase.

**Figure 4:**
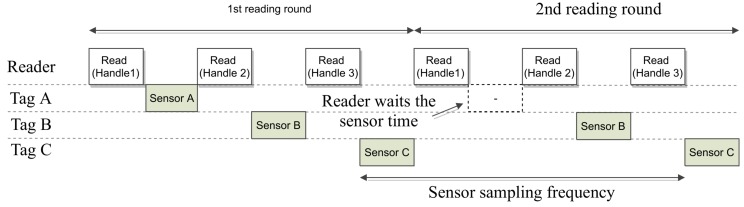
Example of two Read rounds using sensor Frame Slotted Aloha protocol (sFSA) (N=2, S=2).

1. Identification Phase
  This phase starts when the reader transmits the first Query command to identify the *N* tags inside the reader’s range, and it ends when the reader has received one handle from each sensor tag or when a previously determined number of queries have been sent. This fixed number prevents the reader from remaining in the Identification Phase for an undesired long time and not reaching the sense phase. After receiving the EPC, the reader requests the handle at the end of this phase, therefore, the reader ends up with a clear overview of all the tags into its antenna range, their EPCs and handles. The reader commands flow to identify one tag would be as follows (assuming no collision occurred): *Query*-*ACK*(RN16)-*Req_RN16*(RN16).
2. The Sense phase
  It starts when the reader transmits the first Read command. Then, the reader transmits consecutive *Read* commands to cyclically read one set of data from each sensor. This phase ends when the reader has received the required number of sensor reads *S* or after a determined number of Read commands have been sent. As well as in the Identification Phase, this fixed number prevents the reader from remaining in the sense phase for an undesired long time, and keeps the reading rounds time underneath an upper bound.
  For instance, Figure 4 assumes N=3 and S=2, and the Sense phase would consist of the following reader commands flow: *Read*(handle 1)-*Read*(handle 2)-*Read*(handle 3) for 1 read round; the next round is similarly repeated. This procedure needs the reader to associate these handle strings to each EPC, and the tags to remember their handle until the Sensing phase is over.
  Moreover, the isolation of the reading round from the inventory round brings several benefits. The sensor sampling frequency increases with respect to other anti-collision protocols used for sensing, such as the EPC C1G2. Furthermore, the main advantage is that once the reading round has started, this sensor sampling frequency is kept during the whole reading round. That is, the measurements received are time equidistant which allows the usage of these sensors for industrial purposes or streaming applications.
  It can be also seen in Figure 4 that on the second round, tag A does not transmit into its slot. However, the reader waits for the time booked for the transmission of tag sensor A until it transmits a new Read command. This facilitates the streaming of sensors using constant intervals.

**Figure 5:**
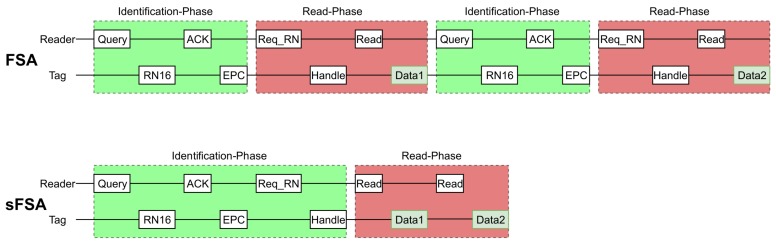
Comparison of the streaming procedure using the standard EPC C1G2 on top of the figure, and the proposed approach below for N=1 and S=2.

Figure 5 shows a comparison of both the EPC C1G2 procedure and the custom protocol. It can be illustrated that in the standard EPC C1G2, in order to receive additional sensor data, the reader must receive the EPC code and one handle from a sensor tag before transmitting a Read command. The custom protocol improves the efficiency of sensors data streaming, by avoiding the overhead caused by having to identify again each sensor tag before reading additional sensor data. Additionally, the EPC C1G2 procedure does not allow to meet constant or quasi-constant sensor sampling frequencies since, every measurement received is subjected to the Identification Phase. That is, tags’ collisions will affect not only the process of tags’ identification but also the process of reading the sensors’ data. Therefore, any additional sensor data read will have a certain probability of tags collisions. In the proposed protocol, collisions only affect the Identification Phase (assuming that every tag in the system chooses a unique handle). Once tags are identified, the reader can read consecutive sensor data without experiencing tag collisions.

### The Problem of the Handles’ Similarities

The sFSA takes advantage of storing the tag handle produced after receiving the unique EPC to cyclically read a particular tag. The EPC has a usual length of 128 bits, and the length of the handle is 16 bits. The total number of distinguishable tags with the EPC is highly superior than with the handle code ($2^{16}=65535$ tags $<<2^{128}$ tags). Thus, the probability of assigning the same handle to two different tags is higher. It is, thus, affordable to use a fixed handle in a local RFID sensor network since the probability of having two tags under the same handle is very small. Nevertheless, in case that could happen, a methodology to repeat the process of the handle reassignment to a tag has been included.

**Figure 6:**
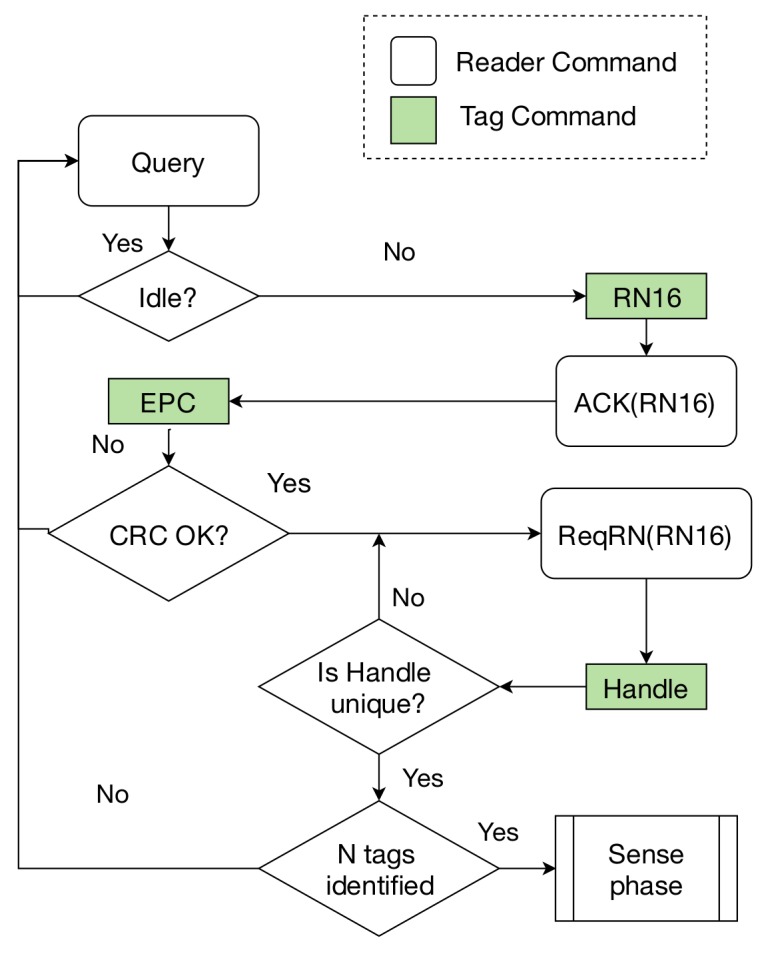
Identification Phase of sFSA using the unique handle procedure.

The Identification Phase of the sFSA is shown in Figure 6. After identifying the type of slot received (idle, collision or success), the reader starts the process of receiving the handle code. It can be observed that whenever a new handle is received, the reader checks the uniqueness of the most recent handle comparing it with a database of the already received handles. In case it matches with any of the already stored ones, the reader transmits a new *ReqRN* command requesting a new handle value. This procedure assures the singularity of all the handles the reader is working with.

## 4. Implementation of the Sensing Protocols

To validate the efficiency of the sFSA, an RFID sensor network for custom sensor data streaming has been developed with an SDR UHF RFID reader and accelerometer sensors included in WISP tags (version 5.1). Because this reader is software-defined, sFSA and EPC C1G2 can be implemented by writing user-level software in C++.

### 4.1. Used Hardware

**Figure 7:**
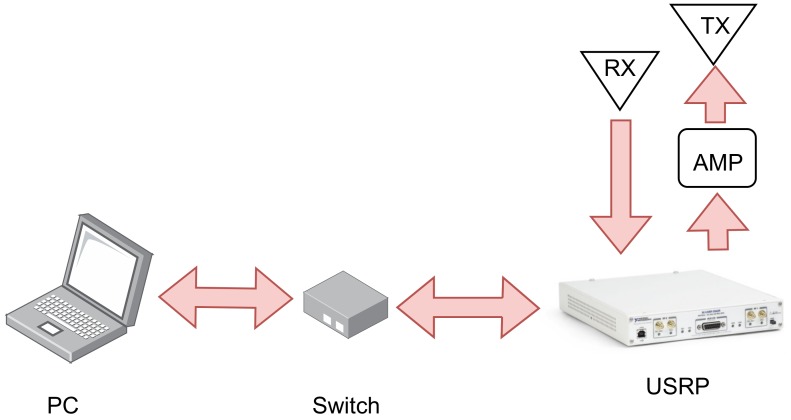
Flow diagram to differentiate the types of tag responses.

This work presents an SDR-RFID reader with a single SBX daughter-board, the USRP N210, capable of communicating with WISP tags (version 5.1) and obtaining the data from their attached sensor; connected to a PC with Linux, where all processing of the backscattered signal is performed, the executions are launched and the data is collected. To communicate the PC with the USRP, a Gigabit data switch has been used. This, allows us to get data at high rates in order to avoid possible overflows. The transmit and receive ports of the daughterboard are connected to two circularly polarized patch antennas of 6 dBi gain. In order to increase the transmission antenna’s power, an HMC453QS16G amplifier, whose gain is typically 21.5 dB at 0.4 GHz and 8 dB at 2.1 GHz has been included between the USRP TX line and one of the patch antennas. The schematic of the hardware system used is presented in Figure 7.

The reader uses the software modules of the system presented in [19], built on GNU radio 3.7.11 (source, matched filter, gate, decoder, and reader), with extended functionalities, highlighting the slot classification procedure, the anti-collision capabilities, and the implementation of additional reader commands.

### 4.2. Slot Classification Procedure

**Figure 8:**
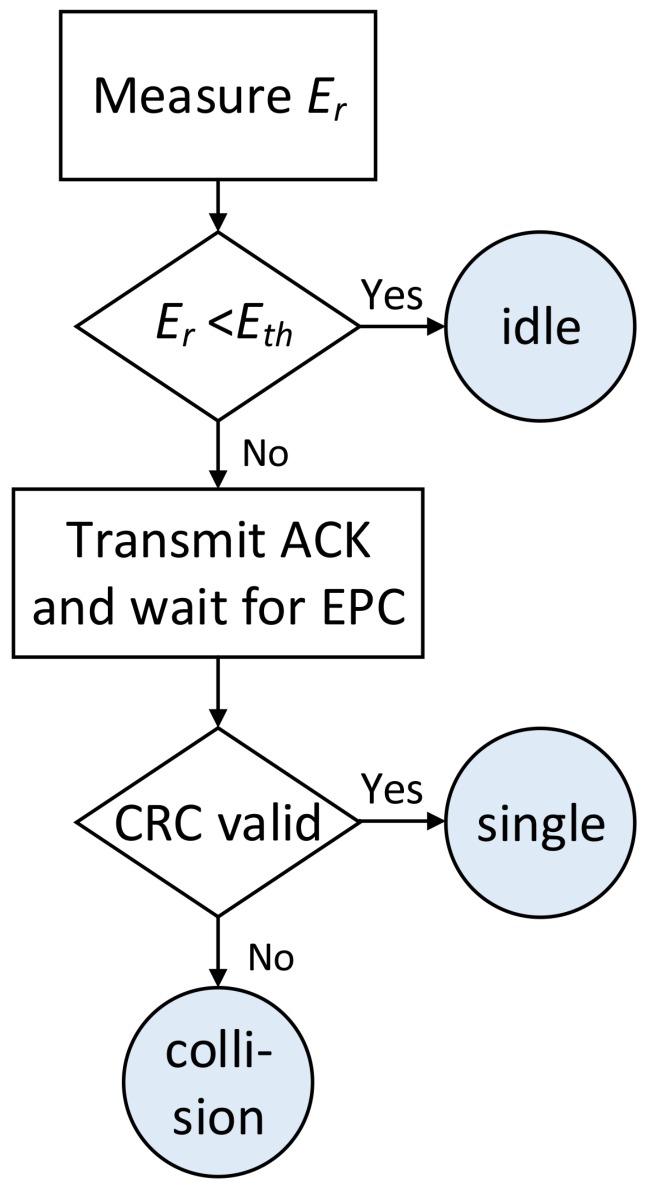
Flow diagram to differentiate the types of tag responses.

In order to differentiate types of slots, a threshold energy value, $E_{th}$, is set for the energy of the received signal. First, an idle or a responded slot is sought. An idle slot occurs when the energy of the received signal is lower than $E_{th}$. The next step consists of determining if there is a collision. The USRP detects it using the CRC attached to the received EPC; thus, the reader is always forced to send an ACK after detecting a signal higher than $E_{th}$. After a correct CRC, a successful slot is detected. Otherwise, the received message is interpreted as a collision. This process is shown in Figure 8.

### 4.3. Anti-Collision Capabilities and Additional Reader Commands

**Figure 9:**
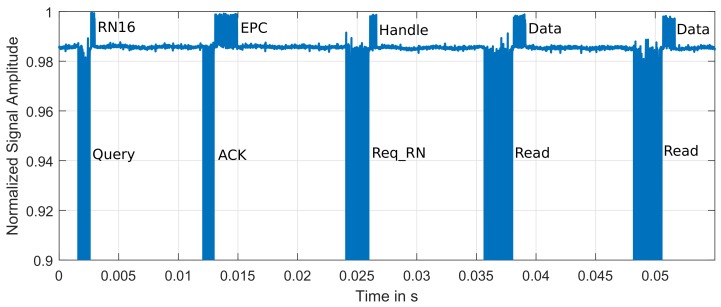
Three consecutive Read commands and three Wireless Identification and Sensing Platform (WISP) accelerometer tag responses.

The SDR reader employs FM0 with Tari = 12.5 us for the commands transmissions, and it has been configured to decode WISP data transmitted with a backscatter-link-frequency, BLF, of 160 Khz. The reader transmission frequency is set to 915 MHz. An example of the Sense phase of the custom protocol for three different WISP accelerometer tags using the custom protocol is shown in Figure 9. This figure shows one accelerometer sensor read for each one of the three tags.

The WISP tags have been reconfigured to store their last transmitted handle until they lose their state. Each accelerometer sensor dataset consists of 32 bits organized in two 16-bit words. Therefore, in order to request one dataset from a WISP tag, the Read command’s *Word-count* has to be set to 0b00000010. The WISPs collect the data from their accelerometer sensor, save it in the user memory and update it periodically.

## 5. Performance Analysis

In this section, an experimental evaluation and comparison of the proposed protocol and the EPC C1G2 are presented. The experiments were carried out using the previously explained hardware setup with a different amount of WISP tags positioned in the field of the reader. With an increasing number of WISP tags, the collisions and interferences have a strong impact on the performance of this evaluation platform, such that no more than N=5 WISP tags could have been used. The SDR implementation of the protocol was adapted in a way that a timer was started, as the first Query command is transmitted, and stopped whenever the requested number of data sets from the sensor tags was received. A second timer was used to measure the time needed for the Identification Phase only. All measurements shown in the following paragraph use the median as the statistical measure to represent the measured data from a minimum of 30 successful executions. For a given number of tags within the range of the reader, *Q* was set to the optimized value, according to [11].

**Figure 10:**
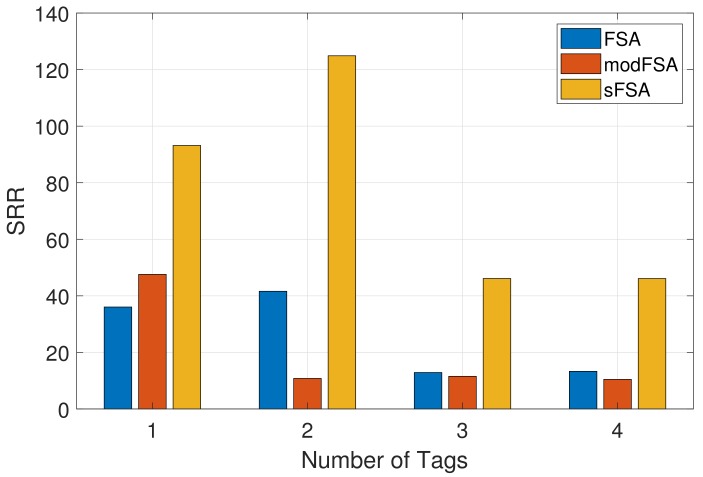
Comparison of SRR for the Framed Slotted Aloha (FSA), the modified FSA and the sFSA protocols for *N* = 1, 2, 3 tags and *S* = 5 reads per tag.

Figure 10 shows a comparison of the SRR for the FSA and the sFSA protocol. The SRR was measured for a given number of tags N=1,2,3,4 positioned within the reader’s range. As the FSA protocol does not store any of the EPCs, consecutive reads can be from the same or a different tag and thus, for a predefined number of reads per tag *S* = 5 all 20 reads, considering *N* = 4 could be from the same tag. To allow for a more comparable measurement, an additional modified FSA (modFSA) protocol was developed. This protocol stores the EPCs after the Identification Phase and thus allows the reader to obtain sensor data from every tag in the field of the reader before reading a particular tag for the second time. As expected, the results for *N* = 1 show, that FSA and modFSA present a very similar behaviour, as no EPCs have to be saved at this point. The increase in the SRR achieved by the sFSA protocol can be explained by the only once executed Identification Phase which is followed by *S* = 5 consecutive reads as sketched in Figure 5 for *S* = 2. With an increase of the number of tags, the number of overall reads also increases, which results in the sFSA outperforming the FSA and the modFSA, as the ratio between the executed time used for the Read Phases and the Identification Phases increases.

**Figure 11:**
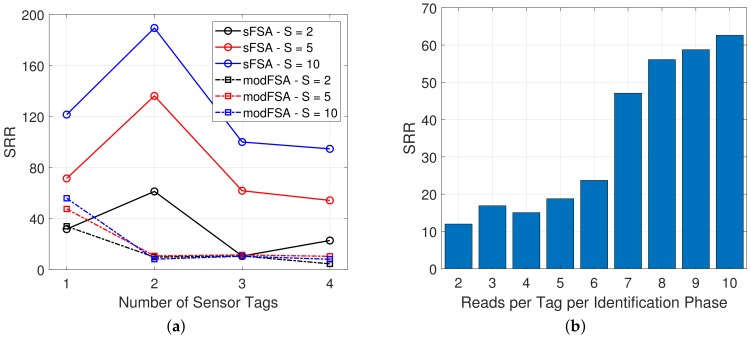
Results of the comparison between the modified FSA (modFSA) and the sFSA protocol with varying tags and reads per tag. (**a**) Comparison of SRR between the modFSA and sFSA with *N* = 1, 2, 3, 4 tags and *S* = 2, 5, 10 reads per tag. (**b**) Variation of the number of reads per tag in the Sense phase with *N* = 3.

The same effect as with an increasing number of tags can be observed increasing the number of reads per tag. Figure 11a shows a comparison of the modFSA and the sFSA protocol for *S* = 2, 5, 10 reads per tag. As the modFSA protocol uses a complete Identification Phase previous to every Read Phase, the increase in the number of reads per tag does not affect the SRR. Thus, the performance of the sFSA protocol considering the SRR increases significantly with an increase of *S*, the number of reads per tag.

A further experimental evaluation of the influence of the number of reads per tag on the SRR has been carried out using the sFSA protocol and three WISPs positioned in the reader’s range. Figure 11b shows the measured data obtained by increasing the *S* from two to 10. A significant rise of the SRR with an increase of *S* is visible. This behaviour is due to the Identification Phase being executed only once, and thus, consecutive reads being executed rapidly after the identification has ended.

In addition, to provide further details on the complexity of the sFSA, Table 1 is included to show the average number of slots needed to perform one read of a sensor using modFSA and sFSA. These results confirm that sFSA needs fewer slots to obtain one sensor data than modFSA at the expense of having to store and compare the EPCs to switch to the Reading phase when all tags have been identified. However, identifying all the tags to read them is a desirable behaviour, and also to perform a fair comparison between the two protocols, modFSA should go through the same process of storing the EPCs to know if all the existing tags in the reading range have been identified. Considering this, the differences between sFSA and FSA become higher with a higher *N*, which highlights the benefits of sFSA when using a larger *N*. Thus the strategy used by sFSA of separating Identification and Reading phases shows a promising performance. It can be inferred from these results that increasing *S* would involve less cost for sFSA than for FSA, even in large *N* groups, and that is confirmed by Figure 11b.

**Figure 12:**
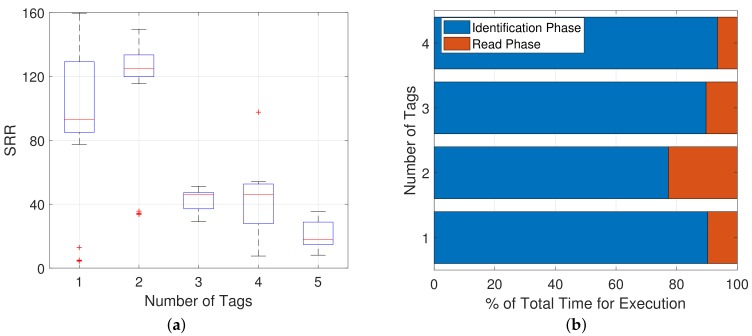
sFSA results on SRR with *N* = 1, 2, 3, 4 and *S* = 5. (**a**) Boxplot of sFSA with *N* = 1, 2, 3, 4, 5 and *S* = 5. (**b**) Percentage of Identification and Sense Phase of the total time execution with *N* = 1, 2, 3, 4, 5 and *S* = 5.

In Figure 12a, a boxplot of the SRR’s of the sFSA protocol for a different numbers of tags *N* = 1, 2, 3, 4, 5 is presented. It can be seen that the read rate decreases radically with the number of tags placed in the environment. This fact is due to the Identification Phase being part of the calculation process. The Identification Phase elongates with more tags in the field of the reader, as more and more collisions and idle slots will appear. Additionally, the pre-defined frame size will not always match the number of tags present.

Figure 12b shows the percentage of the tag sensing procedure dedicated to the Identification Phase (Id.) or to the sensing phase (Sense) using the sFSA protocol. It can be observed that the larger the number of tags, the lower the percentage of the sensing phase. This means that the Identification Phase, which is the most time consuming task, becomes the main bottleneck.

### Discussion and Identified Limitations

The proposed sFSA protocol is a very time efficient solution to obtain sensor data from an RFID sensor tag. Nevertheless, the protocol still relies on the knowledge of knowing the number of tags placed within the range of the reader. The proposed modification, thus, provides a very promising performance for streaming scenarios where sensor tags are fixed and only reading their sensor values is needed. As it has already been discussed, the disappearance of a tag during the reading phase would not affect the reading round, except for the particular sensor not being read, since the reader keeps the sensor sample frequency regardless of the tag responses. On the other hand, for the case where a new tag appears in the reading range of the reader, this scenario represents a new challenge for the sFSA. At this point, sFSA will ignore the appearance of a new tag whilst the reader is executing a reading round. Thus, it will not notice this appearance until a new inventory round is executed. This dynamic scenario with arriving and leaving tags could be handled by upgrading the anti-collision protocol to a dynamic frame size aloha protocol, but the deterministic feature of knowing the exact number of tags in the range would be lost. Therefore, the inherent uncertainty of reading all the sensors or tags in the reader’s antenna range of all RFID scenarios would always exist.

The protocol was experimentally tested for a small number of tags N=5. As RFID sensor networks are assumed to use a number of tags below N=10, the behaviour is expected to be as shown in the performance evaluation. Considering the ETSI region, radio regulations limit the amount of time a single channel shall be used to four seconds. After this time window a break of 100 ms is required before transmitting again on the same channel. This restriction limits the time available for one execution of the proposed protocol and thus limits the number of tags as well as the number of reads per tag. For a typical RFID sensor network scenario with the number of tags not exceeding N=100, this regulation is expected not to be limiting up to at least 100 reads per identification phase.

## 6. Conclusions and Future Work

A high read rate protocol, named sFSA, to stream data from an RFID sensor network is presented in this work. The EPC C1G2 standard protocol, using an optimal frame size for the number of tags on the reader’s read range, results in a very poor performance when reading the sensor data from several different tags. A custom protocol, sFSA, which improves the SRR by more than five times on average with respect to the standard EPC C1G2, is presented. These results have been obtained using a validation hardware setup composed of an SDR reader and up to five WISP CRFID tags. The final results show that the proposed sFSA is a suitable candidate to perform high read rate streaming operations where a constant sensor sampling frequency is sought.

The experimentation performed determines possibilities of further improving the proposed sFSA to work on real environments where the number of tags is unknown. The experiments presented in the paper have been performed using fixed sets of tags and frame sizes adapted to the particular size of each set. The Sensing phase was launched whenever the tag set was fully identified. The reader, however, could be updated to use a dynamic FSA, to adapt to the particular number of tags in the reading range. Then, the Identification Phase would start with a predefined number of inventory rounds and after finishing all of them, the Sense phase would start for the previously identified number of tags. 

## Figures and Tables

**Table 1:** Comparison of the number of slots needed to read one tag between the modFSA and the sFSA.

*N*	modFSA (Slots/Read)	sFSA (Slots/Read)
*1*	6.1	5.74
*2*	46.23	2.16
*3*	79.13	31.47
*4*	118.16	29.96
